# Structure and function of the transketolase from *Mycobacterium tuberculosis* and comparison with the human enzyme

**DOI:** 10.1098/rsob.110026

**Published:** 2012-01

**Authors:** Elizabeth Fullam, Florence Pojer, Terese Bergfors, T. Alwyn Jones, Stewart T. Cole

**Affiliations:** 1 Global Health Institute, EPFL, 1015 Lausanne, Switzerland; 2 Department of Cell and Molecular Biology, Uppsala University, 751 24 Uppsala, Sweden

**Keywords:** transketolase, *Mycobacterium tuberculosis*, X-ray crystallography, pentose pathway, enzyme kinetics

## Abstract

The transketolase (TKT) enzyme in *Mycobacterium tuberculosis* represents a novel drug target for tuberculosis treatment and has low homology with the orthologous human enzyme. Here, we report on the structural and kinetic characterization of the transketolase from *M. tuberculosis* (TBTKT), a homodimer whose monomers each comprise 700 amino acids. We show that TBTKT catalyses the oxidation of donor sugars xylulose-5-phosphate and fructose-6-phosphate as well as the reduction of the acceptor sugar ribose-5-phosphate. An invariant residue of the TKT consensus sequence required for thiamine cofactor binding is mutated in TBTKT; yet its catalytic activities are unaffected, and the 2.5 Å resolution structure of full-length TBTKT provides an explanation for this. Key structural differences between the human and mycobacterial TKT enzymes that impact both substrate and cofactor recognition and binding were uncovered. These changes explain the kinetic differences between TBTKT and its human counterpart, and their differential inhibition by small molecules. The availability of a detailed structural model of TBTKT will enable differences between human and *M. tuberculosis* TKT structures to be exploited to design selective inhibitors with potential antitubercular activity.

## 2. Introduction

*Mycobacterium tuberculosis* is the aetiologic agent of tuberculosis (TB), a disease that is one of the leading causes of death from a single infectious agent worldwide. The World Health Organization currently estimates that 1.8 billion people, approximately one-third of the world's population, are infected with *M. tuberculosis*, and that there are 9 million new active cases annually and 2 million deaths each year as a result of infection [1]. TB treatment is complicated, requiring at least three drugs, of long duration and often accompanied by side-effects. This has resulted in poor compliance to treatment regimens that, in turn, have contributed to the emergence of numerous multi-drug-resistant (MDR) and extensively drug-resistant (XDR) strains that further complicate the therapy. In the case of XDR TB, there are usually no effective therapeutic agents remaining to constitute a successful combination therapy regimen [2]. In conjunction with HIV, this represents a serious problem. Therefore, there is an urgent need for the identification of novel targets and pathways within *M. tuberculosis* in order to develop new chemotherapeutic agents.

Analysis of the genome sequence of *M. tuberculosis* H37Rv [3], together with saturation transposon mutagenesis by *Himar*1 [4], has led to the identification of a number of different proteins and biosynthetic pathways, which may be attractive targets for antitubercular therapy as they are predicted to be essential for the survival of *M. tuberculosis in vitro*. One such pathway that has been identified as essential for the survival of *M. tuberculosis in vitro* is the pentose-phosphate pathway (PPP). Furthermore, evidence that this pathway is an important metabolic process for mycobacteria is provided by its conservation in *Mycobacterium leprae,* as this obligate pathogen has undergone massive gene decay, resulting in a core set of genes that are required for its survival in humans [5]. A putative functional transketolase (TKT) gene product, which is part of this non-oxidative branch of the PPP, has been identified in *M. tuberculosis* through a number of proteomic studies and two-dimensional liquid chromatography–mass spectrometry studies [6–10].

**Figure 1.**
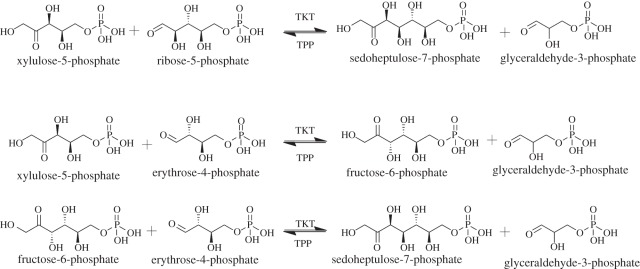
Transketolase enzyme catalysed reactions. TKT catalyses the cleavage of carbon–carbon bonds to transfer two ketol carbon units from donor ketose sugars, like xylulose-5-phosphate, to acceptor aldose sugars, such as ribose-5-phosphate or erythrose-4-phosphate, resulting in the production of sedoheptulose-7-phosphate or fructose-6-phosphate.

TKT enzymes (EC2.2.1.1) have been identified and studied in several organisms, including humans [11,12], *Saccharomyces cerevisiae* [13–15], *Escherichia coli* [16], maize [17], spinach [18] and *Plasmodium falciparum* [19], the causative agent of malaria. These are typically cytosolic enzymes have a molecular mass of 70–75 kDa, with the homodimer being the active entity. This class of enzyme uses the cofactor thiamine pyrophosphate (TPP) and a divalent metal cation to catalyse the cleavage of carbon–carbon bonds to transfer two ketol carbon units from donor ketose sugars, such as xylulose-5-phosphate, to acceptor aldose sugars, such as ribose-5-phosphate or erythrose-4-phosphate, resulting in the production of sedoheptulose-7-phosphate or fructose-6-phosphate, respectively (figure 1) [20–23]. The reaction proceeds via a Ping Pong Bi Bi mechanism. A broad range of donor and acceptor substrates have been reported, with the bacterial, plant and yeast enzymes having a wider range of substrate recognition than human TKT enzymes [22].

The first TKT structure that was reported in literature was from *S. cerevisiae* (pdb 1trk) [24]. Subsequently, a number of other structures have been solved, both with and without sugar substrates and cofactor, for other species including *E. coli* (pdb 2r5n) [25], maize (pdb 1itz) [17], *Leishmania mexicana* (pdb 1r9j) [26], *Thermus thermopilus* (pdb 2e6k)*, Bacillus anthracis* (pdb 3hyl) and *Francisella tularensis* (pdb 3kom). Until recently, no mammalian TKT structures were available; however, the human structure has recently been solved (pdb 3ooy and 3mos) [11]. All of the TKT structures from the different species show a similar overall TKT fold and arrangement of three domains [13,17,24,26,27]. Domains I (1–322) and II (323–527), numbering from the TKT enzyme from *S. cerevisiae*, have been shown to be involved in dimeric interactions of each monomeric subunit and are also involved in TPP cofactor binding and recognition. The third domain, which comprises the last approximately 150 amino acids, is believed to be involved in the regulation of the activity of the enzyme and in stereochemical control of the sugar substrates, in which D-threo at the C-3 and C-4 positions are favoured [13,24,28–30]. Studies have shown that the TKT enzyme from *E. coli* is still active in the absence of the third domain [28].

Despite its importance in plants and bacteria, detailed studies of TKTs from a mycobacterial species have not yet been reported. The cell wall of *M. tuberculosis* is unique in its complexity, comprising three covalently attached layers (peptidoglycan, arabinogalactan and mycolic acids), and is very important to the survival of *M. tuberculosis*, its pathogenicity and impermeability to drugs. One of the first committed steps in the production of the arabinogalactan layer of *M. tuberculosis* is the production of D-ribose-5-phosphate. The biosynthesis of D-ribose-5-phosphate can potentially occur through two processes [31]: either the enzyme ribose-5-phosphate isomerase (Rv2465) can convert ribulose-5-phosphate to D-ribose-5-phosphate [32], or the TKT enzyme (Rv1449) can convert sedoheptulose-7-phosphate to D-ribose-5-phosphate. Given that the ribose-5-phosphate isomerase enzyme Rv2465 is not essential based on the studies of Sassetti *et al*. [4], it is thought that the TKT plays a key role in linking the non-oxidative part of the PPP to biosynthesis of pentose sugars and hence to essential arabinans in cell wall biosynthesis. A number of current antitubercular drugs target key components of the cell wall, including isoniazid and ethionamide (which block mycolic acid synthesis [33–35]), ethambutol (which targets arabinogalactan formation [36]) and cycloserine (which inhibits the biosynthesis of peptidoglycan [37,38]). Recently, a new class of compounds, 1,3-benzothiazin-4-ones, have been identified that prevent the formation of arabinose by inhibition of the decaprenyl-phosphoribose epimerase activity catalysed by the DprE1 and DrpE2 proteins [39].

Given the potential importance of TKT in the production of arabinan in *M. tuberculosis,* this biosynthetic pathway is an attractive target for identifying additional, novel antitubercular agents. As the first step in this process, we report here our structural and functional studies of the TKT enzyme from *M. tuberculosis* (TBTKT), encoded by gene *rv1449c,* and discuss structural differences between the human, yeast and bacterial homologues.

## 3. Material and methods

All chemicals and reagents were purchased from Sigma-Aldrich, unless otherwise stated. Restriction enzymes were obtained from New England Biolabs. Double-distilled water was used throughout.

### 3.1. Plasmid construction

The putative TKT gene, *rv1449c*, was amplified in two steps by PCR from cosmid I392 of *M. tuberculosis* using gene-specific primers, followed by gateway adaptor primers with attB1 and attB2 sites for incorporation into the gateway entry vector pDONR207 (Invitrogen). The gateway adaptor forward primer encoded a thrombin recognition sequence ctggttccgcgtggatc. The first PCR step used primers 5′CTGGTTCCGCGTGGATCCACCACACTCGAAGAGATCTCCG (forward) and 5′-CAAGAAAGCTGGGTCTCAGTTATCCAGCGCTCGTTCG-3′ (reverse). The second PCR reaction used primers 5′-ggggACAAGTTTGTACAAAAAAGCAGGCTTCctggttccgcgtggatc-3′ (forward) and 5′-ggggACCACTTTGTACAAGAAAGCTGGGTC-3′ (reverse). For both PCR reactions, PCR amplification consisted of 30 cycles (95°C, 2 min; 95°C, 1 min; 60°C, 30 s; 72°C, 3 min), followed by an extension cycle (10 min at 72°C). The resulting PCR product was cloned into the pDONR207 vector and the resultant plasmid was used to transfer the gene sequence into pET160_DEST (Invitrogen; N-term hexa-histidine-tag) by homologous recombination. The Rv1449c_pET160_DEST plasmid obtained was sequenced fully and used for protein expression.

### 3.2. Heterologous overexpression of transketolase enzyme from *Mycobacterium tuberculosis*

*Escherichia coli* BL21(DE3) transformed with the Rv1449c_pET160_DEST plasmid was grown at 27°C to an optical density at 600 nm (OD_600_) of 0.6–0.8 in 2xLuria-Bertani (LB) medium supplemented with 100 μg ml^−1^ ampicillin. The production of the protein was induced with 500 μM isopropyl-β-thiogalactopyranoside (IPTG) and the cultures were grown at 16°C overnight with shaking. The cells were harvested by centrifugation at 6000 g for 20 min at 4°C, and the cell pellet was resuspended in lysis buffer (50 mM sodium phosphate, 500 mM sodium chloride, 2 mM TPP, 10 per cent glycerol, 5 mM β-mercaptoethanol, 0.1% Triton-X 100 and pH 7.4) and Complete Protease Inhibitor Cocktail (Roche). The cells were freeze–thawed and sonicated. Following centrifugation at 18 000 g for 30 min at 4°C, the supernatant was loaded onto a Cobalt-Talon affinity column.

### 3.3. Purification of transketolase enzyme from *Mycobacterium tuberculosis*

Recombinant TBTKT was purified in three steps. The soluble lysate was incubated with Talon-resin (Clontech) at 4°C for 1 h. The column was washed with 20 mM sodium phosphate, 100 mM sodium chloride, 2 mM TPP, 10 per cent glycerol, 5 mM β-mercaptoethanol (pH 7.4) and the His-tagged protein eluted with increasing concentrations of imidazole. The 50 and 250 mM imidazole fractions as determined by SDS-PAGE were pooled, concentrated (Amicon centrifugal device) and run on a Resource Q column (GE Healthcare) in 20 mM sodium phosphate, 100 mM sodium chloride, 2 mM TPP and 5 mM β-mercaptoethanol (pH 7.4), and eluted with sodium chloride (0.1–1 M). Fractions containing TBTKT were pooled and purified further using size exclusion chromatography. Gel filtration experiments were carried out on a Superdex 200 16/60 column (GE Healthcare). The gel filtration column was run in 20 mM sodium phosphate, 100 mM sodium chloride, 2 mM TPP, 5 mM β-mercaptoethanol (pH 7.4). Fractions containing TBTKT were pooled, and the dimer and monomeric fractions collected separately.

### 3.4. Crystallization of transketolase enzyme from *Mycobacterium tuberculosis*

Monomeric TBTKT was concentrated to 8 mg ml^−1^ (Amicon ultracentrifugal concentrators from Millipore) and buffer exchanged into 20 mM Tris-HCl, 1 mM ethylenediaminetetraacetic acid **(**EDTA), 1 mM dithiothreitol (DTT) (TED) buffer plus 5 mM MgCl_2_, 5 mM TPP (pH 7.6) or 20 mM glycyl-glycine (Gly-Gly), 5 mM MgCl_2_, 10 mM TPP (pH 7.7). The crystals were grown using the sitting-drop vapour-diffusion technique by mixing equal volumes (150 nl) of concentrated TBTKT with mother liquor using a protein crystallization robot (Mosquito, TTP LabTech). TBTKT crystals grew within one week at 22°C, in 0.1 M ammonium acetate, 0.1 M bis-tris pH 5.5 and 17% w/v polyethylene glycol 10 000 with the protein in buffer 20 mM Gly-Gly, 5 mM MgCl_2_, 10 mM TPP (pH 7.7).

### 3.5. Data collection, structure determination and refinement

The TBTKT crystal was cryoprotected with 30% v/v glycerol and flash frozen in liquid nitrogen prior to data collection. Data were collected on beamline PXIII at the Swiss Light Source (Villigen, Switzerland) with a mar225 mosaic CCD detector. The space group was determined, and the data were integrated, scaled and merged using the program XDS [40]. PHASER [41] was used to solve the TBTKT structure by molecular replacement with a TBTKT model, built using Swiss-Model [42] and the TKT structure from *E. coli* (PDB code 2r8o chain A [25]) as a template. The structure was refined by iterative cycles of alternating manual rebuilding in O [43] and Coot [44], and reciprocal space crystallographic refinement with Refmac5 [45]. The successful refinement was dependent on making use of the non-crystallographical symmetry for averaging using the tools within O. Data processing and refinement statistics are shown in table 1. Preparation of structure-related images was carried out with PyMOL (version 0.99, Schrödinger, LLC). Sequences were aligned with ClustalW2, and sequence figures were generated with ESPript version 2.2 [47,48].

**Table 1.** Data collection and refinement statistics. Numbers in brackets denote values for the highest-resolution shell.

	TBTKT (3RIM)
*data collection statistics*	
beam line	PXIII – Swiss Light Source
space group	*P*_1_
resolution range (Å)	50–2.49 (2.64–2.49)
wavelength	0.979000
cell dimensions	
cell axial lengths (Å)	*a* = 75.5, *b* = 80.1, *c* = 130.0
cell angles (^o^)	*α* = 82.2, *β* = 81.2, γ = 66.4
number of molecules in the asymmetric unit	4
redundancy	3.9 (3.65)
completeness (%)	96.6 (91.2)
*R*_meas_^a^	15.9 (54.0)
mean I/σI	7.90 (2.77)
*refinement statistics*	
resolution range	50–2.49
*R*_work/_ *R*_free_	0.22/0.27
number of atoms	
protein/ligand/water	21206/156/410
Ramachandran	
favoured/allowed/disallowed (%)	88.4/11.6/0
average B-factor (Å^2^)	
protein/ligand/solvent	27.2/27.6/19.3
root mean square deviations	
bond lengths (Å)	0.017
bond angles (Å)	1.634

^a^*R*_meas_ is defined by Diederichs & Karplus [46].

The atomic coordinates and experimental structure factor data of the refined *M. tuberculosis* TKT have been deposited in the protein data bank (PDB code 3RIM).

### 3.6. Steady-state kinetic analysis

Activity of the recombinant TKT was measured by the reduction of potassium ferricyanide by the α-carbanion intermediate formed, as described previously by Kochetov [49]. The reaction was carried out in 100 μl in a 96-well plate, in 50 mM Gly-Gly buffer (pH 7.6), 2 mM magnesium chloride, 0.1 mM TPP, 0.5 mM potassium ferricyanide, 3 mM fructose-6-phosphate (6FP) and recombinant TBTKT enzyme, and the reduction of potassium ferricyanide was monitored at 420 nm on a Tecan Infinite M200 plate reader at 37°C. The *K*_m*,*app_ values were determined by varying the concentration of substrate from 0 to 4 mM. Alternatively, the activity of the recombinant TKT was measured using the auxiliary enzymes: triosephosphate isomerase and α-glycerophosphate dehydrogenase. The reaction (100 μl) was performed in a 96-well plate, in 50 mM Gly-Gly buffer (pH 7.6), 2 mM magnesium chloride, 0.1 mM TPP, 0.4 mM NADH 4 mM ribose-5-phosphate (R5P) and 4 mM xylulose-5-phosphate (X5P), 8 units of triosephosphate isomerase and 8 units of α-glycerophosphate dehydrogenase. Oxidation of NADH was followed spectrophotometrically by measuring the absorbance at 340 nm (A_340_), as described previously by Kochetov [21]. Substrate concentrations were varied to below and above the *K*_m,app_ value. The program Prism (version 5, GraphPad Software, La Jolla, CA, www.graphpad.com) was used for nonlinear regression analysis of kinetic data. Inhibitors oxythiamine (Sigma) and 5-benzyl-3-phenylpyrazolo[1,5-α]pyrimidin-7(4H)-one (Chembridge Screening Library) were added at a final concentration of 30 μM and tested in both enzyme assays.

### 3.7. Testing for *in vitro* growth inhibition of *Mycobacterium tuberculosis*

The inhibitory activity of oxythiamine was tested at a concentration range of 0–100 μg ml^−1^ in the resazurin reduction assay as described previously [39].

## 4. Results

### 4.1. Identification and production of transketolase enzyme from *Mycobacterium tuberculosis*

**Figure 2.**
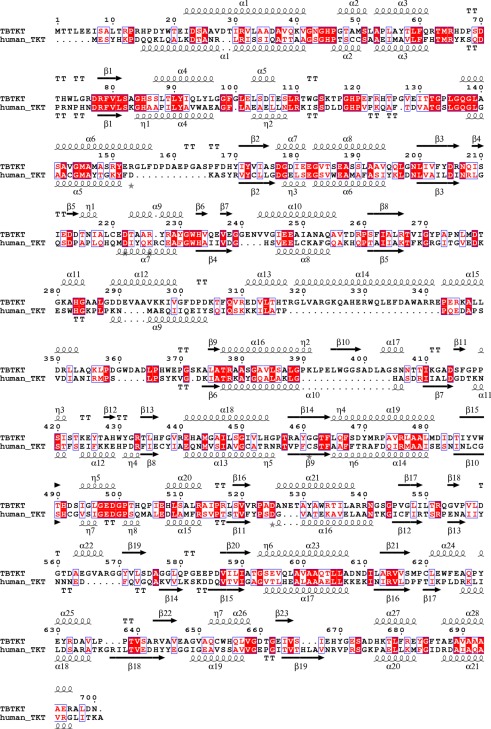
Sequence comparison of transketolase enzyme from *Mycobacterium tuberculosis* (TBTKT) and human transketolase (TKT). Sequence alignment of TBTKT versus human TKT using the programs ClustalW2 and ESPript version 2.2 [47,48]. Numbering corresponds to the sequence of TBTKT. Identical residues are indicated by a red background, and conserved residues are indicated by red characters. The secondary structure elements of TBTKT are shown above the sequences, and those of human TKT are shown below.

A putative TKT gene (*tkt, rv1449c*; hereafter TBTKT) was annotated in the genome of *M. tuberculosis* (http://tuberculist.epfl.ch/index.html) based on the presence of a consensus sequence for TPP binding and extensive sequence similarity to other known TKT genes. The predicted open reading frame of TBTKT contains a specific sequence motif of THDSIGLGEDGPTHQPIE that has been identified previously in other TKT proteins [50] and corresponds to amino acids 490–507 in this enzyme. Interestingly, a second sequence motif, which is common to thiamine cofactor-binding enzymes, is a GDG consensus motif followed by 21 amino acids varying in sequence identity. In *M. tuberculosis*, the GDG motif has been replaced by SDG (amino acids 176–178), and this is also the case in all other sequenced mycobacterial TKTs (figure 2; electronic supplementary material, S1). No other known TKT enzymes that have been studied contain this mutation in the TKT consensus sequence [11].

To produce recombinant TBTKT protein, primers were designed and the full-length *tkt* gene was cloned with an N-terminal hexa-histidine tag and over-expressed in an *E. coli* expression system. Soluble, active protein was obtained in a yield of 10 mg l^−1^ bacterial culture and purified to apparent homogeneity.

### 4.2. Overall structure of transketolase enzyme from *Mycobacterium tuberculosis*

Crystal trials of TBTKT were set down as described (§3.4). Crystals typically grew after 7 days, and diffraction data were collected from crystals that were formed in 17% w/v polyethylene glycol 10 K, 0.1 M ammonium acetate, in 0.1 M bis-tris at pH 5.5. Crystals of the TBTKT were in symmetry group *P1* with four molecules predicted in the asymmetric unit, with a Matthew's coefficient [51] of 2.3 Å^3^ Da^–1^ and a solvent content of 46 per cent. The structure was solved by using molecular replacement with a homology model of TBTKT built using Swiss-Model [42] and the *E. coli* TKT structure (pdb 2r8o) [25] that has 42 per cent amino acid sequence identity to TBTKT. In almost all protein molecules, electron density could be observed for residues 6–700, the last residue of the full-length protein. The final model was refined with tight non-crystallographic restraints to a resolution of 2.5 Å and consists of 2776 amino acids, 410 water molecules, four TPP molecules, 4 Mg^2+^ ions and eight glycerol molecules, with an *R*_work_ of 22 per cent and an *R*_free_ of 27 per cent (pdb 3RIM; table 1).

**Figure 3.**
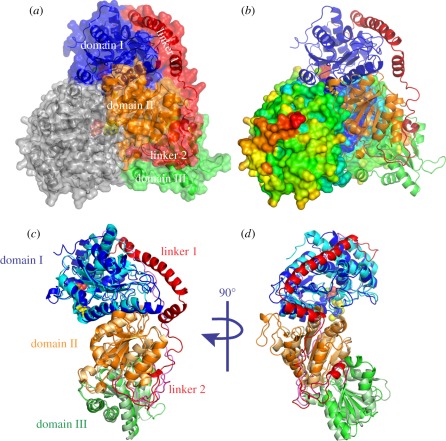
Crystal structure of TBTKT and superposition to human tkt. (*a*) Surface representation of TBTKT dimer with bound cofactor TPP (pink) with one monomer in grey and the second monomer coloured by domains (blue, domain I; orange, domain II; green, domain III; red, linkers 1, 2). (*b*) Dimer representation of TBTKT with one monomer represented as surface and coloured by B-factor, and the second one as a cartoon with the domains coloured as in (*a*). (*c*) Superposition of TBTKT and human tkt (pdb code 3MOS) monomers depicted as cartoon representation. The domains are colour-coded as in (*a*) and (*b*), with human tkt in lighter colours compared with TBTKT. (*d*) The structures are rotated 90° around the twofold axis compared with (*c*).

The TBTKT enzyme forms a homodimer, where the two monomeric units are related by a non-crystallographic twofold axis. There are two dimers per asymmetric unit that are related to each other by a twofold axis to create a non-crystallographic screw axis parallel to the crystallographic *Z*-axis. The overall structure of each TBTKT monomer consists of three domains interconnected by flexible linker regions (figure 3*a*,*b*). The N-terminal pyrophosphate (PP)-binding domain (domain I) that binds the PP part of TPP (residues 6–299) consists of a central five-stranded parallel β-sheet, surrounded by 11 α-helices. The pyridine (Pyr)-binding domain (domain II) that involves the aminopyrimidine moiety of TPP (residues 377–550) comprises a central six-stranded parallel β-sheet with seven α-helices surrounding it. The C-terminal domain (Domain III, residues 571–700) forms a central five-stranded mixed β-sheet with four parallel strands and one antiparallel strand. These three domains are linked by linkers (linker 1: residues 300–376; linker 2: residues 551–570; figure 3*a,b*). Domains I and II are mostly involved in dimer formation, while the third domain makes rather few contacts to the other subunit (figure 3*a*,*b*). The Protein Interfaces, Surfaces and Assemblies (PISA) service at the European Bioinformatics Institute (http://www.ebi.ac.uk/msd-srv/prot_int/pistart.html) gives a complexation significance score of 0.614 between chains A and B (or chains C and D), indicating that the interface plays an essential role in dimer complexation with a large number of residues involved. There are 60 hydrogen bonds and 15 salt-bridges between the two monomers. In total, a surface of around 4200 Å^2^ is buried upon formation of the dimer (chains A and B and chains C and D), with 17 per cent of the total residues being involved in its formation. The solvent accessible surface area of a monomeric subunit is 25 785 Å^2^. TBTKT is also found as a homodimer in solution as seen by size exclusion chromatography (data not shown) and therefore it is likely that the homodimer found in the crystal structure is the biologically relevant unit as shown for other TKT enzymes. Two important conserved residues involved in dimeric interaction are Glu182 and Glu441, the buried acidic side chains of which form a hydrogen bond interaction, allowing Glu441 to interact with the N1′ atom of the aminopyrimidine ring of the TPP [13].

The subunits of different TKT structures were compared and superimposed using DaliLite version 3 [52]. The TBTKT and human TKT structures can be superimposed with a root mean square deviation (RMSD) value of 2.3 Å and a high Dali Z-score of 36.7 (584 Cα atoms were aligned, 23% sequence identity to TBTKT). With other TKTs, the following values were obtained: for yeast (pdb 1trk), RMSD of 1.4 Å, Z-score of 48.4 (678 Cα atoms, 44% sequence identity); for *E. coli* (pdb 2r5n), RMSD of 1.6 Å, Z-score of 48.0 (663 Cα atoms, 42% sequence identity); for *B. anthracis* (pdb 3hyl), RMSD of 1.3 Å, Z-score of 50.1 (663 Cα atoms, 47% sequence identity); for maize (pdb 1itz), RMSD of 1.5 Å, Z-score of 49.4 (666 Cα atoms, 44% sequence identity). The combined superimposition results of TBTKT and other TKT structures confirm the similarity of the overall fold and domain topology of TKT enzymes from different species. However, the TBTKT enzyme is 87 amino acids longer than the human TKT. The deletions in the human TKT correspond to two loop regions at amino acids 153–167 (in domain I) and amino acids 413–432 (in domain II) in TBTKT. No binding is predicted to occur between these residues and substrate or TPP cofactor. In addition, linker 1 is longer and more structured, forming a helix-turn-helix in TBTKT compared with human TKT—77 amino acids (residues 300 to 376) versus 39 amino acids (residues 277 to 315)—and may result in some specificity (figure 3*c*,*d*).

### 4.3. Binding of TPP and Mg^2+^ ion to transketolase enzyme from *Mycobacterium tuberculosis* and comparison with homologues from different species

**Figure 4.**
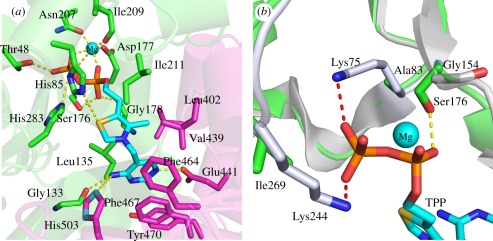
Structure of the cofactor-binding site in TBTKT. (*a*) Illustration showing TPP cofactor as cyan sticks, Mg^2+^ as a cyan sphere as well as selected amino acid residues in stick representation. Amino acids contributed by different monomers are indicated by different colour-coding. (*b*) Superposition of the TBTKT (green) to human TKT (silver) with cofactors TPP and Mg^2+^, and selected residues in stick representation.

Well-defined electron density has revealed the binding pocket of the cofactor TPP and an Mg^2+^ ion in the TBTKT structure. Each TBTKT monomer contains one TPP molecule and one Mg^2+^ ion, suggesting that each active site is equivalent, and this has been found to be the case for other reported structures of TKT enzymes [11,13,15,17,25]. An active site cleft is formed between the two monomeric units allowing the cofactors TPP and Mg^2+^ to bind, such that the N-terminal domain I of chain A binds the PP moiety of TPP, and domain II of chain B interacts with the aminopyrimidine ring. The PP moiety of TPP is anchored in place through a number of hydrogen bonds formed with residues Thr48, His85, Ser176, Asp177, Gly178, Asn207, Ile209 and His283 from one monomer (figure 4*a*). The Mg^2+^ ion is octahedrally coordinated to residues Asp177, Asn207 and Ile209, along with two oxygen atoms of the PP moiety of the cofactor TPP and a water molecule.

Of the eight residues of TBTKT that are involved in hydrogen bonding to the PP moiety of TPP, four residues are conserved among all TKT enzymes, including the mammalian versions, and these correspond to His85, Asp177, Asn207 and His283 in TBTKT. One non-conserved amino acid residue is Thr48, and this is specific to mycobacterial TKTs (electronic supplementary material, figure S1). By superposing the available structures, Thr48 corresponds to an alanine residue in both yeast (pdb 1ngs) [15] and *E. coli* (1qgd) [25], and a leucine in maize (pdb 1itz) [17]; this residue is unable to interact with the cofactor. However, the equivalent serine residue in human TKT (Ser40) also has the potential to hydrogen bond with the terminal PP moiety in a manner similar to that of Thr48 as found in the TBTKT structure. Ile209 is highly conserved among non-mammalian species and is replaced by a leucine residue in human TKT (Leu187), but this does not affect its interaction to the divalent ion through its backbone carbonyl group. The other non-conserved residue that is involved in the binding of the PP segment of TPP is the hydroxyl side chain of Ser176 (figure 4*b*). This is noteworthy since Ser176 represents the mutation in the consensus sequence GDG to SDG. This non-conserved residue in the motif does not affect the overall fold of the protein and forms a turn separating a β-stand from an α-helix in the βαβ-fold. In TBTKT, the hydroxyl side chain is positioned to hydrogen bond with the PP group of the cofactor. However, in the yeast and human structures, the equivalent glycine residues (Gly156 in yeast TKT and Gly154 in human TKT) lack this hydroxyl side chain and cannot form such a hydrogen bond with the PP moiety. The backbone carbonyl oxygen of the glycine residue is pointed away from the cofactor and does not compensate for this lack of hydrogen bond in this way. We demonstrated in our kinetic experiments that mutating GDG to SDG does not affect the activity of the enzyme, and this can be explained at the structural level by the remaining hydrogen bonding of the backbone nitrogen of another residue, Gly178, corresponding to Glu157 in human and Gly158 in yeast, to the PP of TPP. Moreover, additional interactions are formed in human TKT between Lys75 and Lys244 and the PP moiety of TPP that are not present in TBTKT (replaced by Ala83 and Ile269, respectively; figure 4*b*).

The pyrimidine ring portion of TPP binds in a hydrophobic core that is formed by residues from both monomers: Leu135 and Ile211 from one monomer, and Leu402, Val439, Phe464, Phe467 and Tyr470 from the second monomer (figure 4*a*). The pyrimidine ring forms π-stacking interactions with the phenyl ring of residue 467. There are also hydrogen bond interactions between the pyrimidine ring, and Glu441, Gly133 and His503. Glu441 has been demonstrated to be important in activating the cofactor by protonating the N1′ position of the aminopyrimidine ring [13], and it appears that this will also occur in TBTKT. Of these residues, Glu441 and Gly133, as well as Phe464, Phe467 and Leu135, are conserved among yeast and human TKT enzymes. Leu402 and Tyr470 are replaced by slightly more hydrophilic residues in the human equivalent (Thr342 and Arg395, respectively). However, these residues are positioned in a similar way as the yeast TKT and TBTKT residues, and do not form extra hydrogen bonds to the aminopyrimidine ring of TPP.

### 4.4. Kinetic studies of transketolase enzyme from *Mycobacterium tuberculosis*

Apparent kinetic (*K*_m,app_) constants were determined for TBTKT with the two donor substrates—xylulose-5-phosphate (X5P) and fructose-6-phosphate (F6P)—and the acceptor substrate ribose-5-phosphate (R5P). R5P had a *K*_m,app_ of 0.82 ± 0.12 mM, F6P had a *K*_m,app_ of 0.63 ± 0.09 mM and X5P had a *K*_m,app_ of 0.35 ± 0.12 mM (table 2). These kinetic constant values for the TBTKT enzyme are comparable with kinetic constants previously determined for TKTs from *S. cerevisiae* [15,21], spinach [53], *E. coli* [16] and *P. falciparum* [19] (table 2), with X5P having the highest binding affinity. As explained by the crystal structure, mutation of GDG to SDG has no effect upon the TKT reaction as the cofactor TPP still binds to the active pocket in the same manner via additional hydrogen bounds formed (e.g. by Lys75, Lys244 and Gly157 in human TKT). Based on these kinetic studies, it appears that the TBTKT is more similar to the TKTs from bacteria, yeast and plants because it was shown that fructose-6-phosphate is a poor substrate for the human TKT enzyme, with a *K*_m,app_ of 7 mM [22], whereas in TBTKT, F6P is a good substrate for catalysis, with a *K*_m,app_ of 0.63 mM (table 2).

**Table 2.** Steady-state kinetic constants, K_m,app_ (mM), of TBTKT and other transketolase enzymes. Assay conditions for TBTKT are detailed under ‘§2’. n.d., not determined.

	R5P	F6P	X5P	reference
*M. tuberculosis*	0.8 ± 0.1	0.6 ± 0.1	0.4 ± 0.1	this study
human	0.61 ± 0.36	7 mM	0.30 ± 0.79	[11], [22]
*S. cerevisiae*	0.4	1.8	0.21	[21]
*S. cerevisiae*	0.15 ± 0.21	n.d.	0.70 ± 0.10	[15]
*S. cerevisiae* H481A	0.15 ± 0.50	n.d.	1.24 ± 0. 90	[15]
*S. cerevisiae* H481Q	n.d.	n.d.	4.08 ± 0.51	[15]
spinach chloropasts	0.33	n.d.	0.06	[53]
*E. coli*	1.4	1.1	0.16	[16]
*P. falciparum*	n.d.	2.25 ± 0.5	n.d.	[19]

Previous extensive mechanistic studies of TKT enzymes have shown that this class of enzyme follows Ping Pong Bi Bi reaction kinetics where the donor and acceptor substrates are not able to bind to the protein simultaneously [14,22,25,54,55].

**Figure 5.**
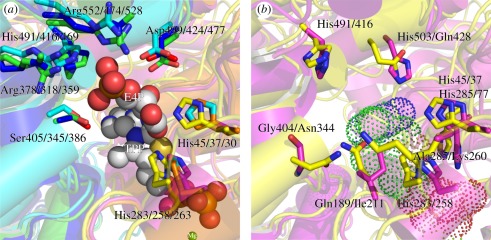
Superposition of active sites of TBTKT (pdb code 3RIM), human TKT (pdb code 3MOS) and yeast TKT (pdb code 1ngs). (*a*) Illustration of the three structures with sphere representation of substrate erythrose-4-phosphate (E4P) and cofactors TPP and Mg^2+^ as found in the active site of yeast TKT. Amino acid residues (amino acid numbering: TBTKT/human TKT/yeast TKT) involved in the binding of E4P to yeast TKT (light blue orange) as well as the corresponding TBTKT (green and pink) and human TKT (dark blue/yellow) amino acid residues are shown in stick representation. (*b*) Illustration of TBTKT and human TKT with selected amino acid residues of TBTKT (magenta) and human TKT (yellow). Cofactors TPP and Mg^2+^ are represented as spheres. Same orientation as in (*a*).

The structure suggests that this would also be the case for TBTKT because the substrate cleft is not large enough to allow binding of donor and acceptor sugar substrates simultaneously. The yeast TKT enzyme has been co-crystallized with the substrate erythrose-4-phosphate (pdb 1ngs) [56], with no large conformational changes observed upon binding of the substrate, and this led to the identification of a number of catalytically important residues for substrate recognition. These residues are all conserved and correspond to His45, His283, Arg378, Ser405, His491, Asp499 and Arg552 in TBTKT (figure 5*a*). The residues Arg378, Arg552 and His491 have been shown to be important in the recognition of the phosphate moiety of the sugar [56]. These residues in TBTKT can be superimposed upon those from the yeast structure and human structure (figure 5*a*), and it is envisaged that the TKT sugar substrates for the TBTKT enzyme are able to bind in a similar manner, and the enzyme reacts via a Ping Pong Bi Bi reaction mechanism [29,30,57,58].

Two inhibitors of the human TKT were tested for inhibitory activity against purified TBTKT enzyme, as well as on live *M. tuberculosis* cells. Oxythiamine is an analogue of the cofactor TPP and inhibits TKT activity in human tumour cells *in vivo* [59], although it is only a weak inhibitor *in vitro* [60]. Oxythiamine has also been found to inhibit weakly the TKT enzyme from *P. falciparum* but to have *in vivo* activity against *P. falciparum* [19]. The other inhibitor of human TKT—5-benzyl-3-phenylpyrazolo[1,5-α]pyrimidin-7(4H)-one (5BPPO), a compound identified from a high-throughput screen of 64 000 compounds against human TKT—was also found to be active against three cancer cell lines, and had IC50 values in the low micromolar range [60]. Oxythiamine had no inhibitory activity on the TBTKT enzyme at a relatively high concentration of 30 µM (table 3). Instead, oxythiamine, at this concentration, increased the rate of the reaction of the TBTKT enzyme by 30 per cent under the assay conditions tested. We are unable to explain this result structurally because oxythiamine is predicted to bind in the same position as TPP in both the TBTKT and human TKT enzymes (results not shown). In addition, 5BPPO also had no inhibitory effect on the activity of TBTKT at a concentration of 30 µM (table 3), which is higher than the IC_50_ value of 4 µM determined for the human TKT enzyme [60]. When tested for their inhibitory effects on the growth of *M. tuberculosis in vitro*, both compounds (oxythiamine and 5BPPO) displayed no growth inhibition at concentrations as high as 100 µg ml^−1^, corresponding to a molar concentration of 332 µM for oxythiamine and 227 µM for 5BPPO (table 3).

**Table 3.** Effects of human TKT inhibitors on TBTKT and *in vitro* growth of *M. tuberculosis*. Assay conditions are detailed under §2. 5BPPO, 5-benzyl-3-phenylpyrazolo[1,5-α]pyrimidin-7(4H)-one.

inhibitor	structure	IC_50_ TBTKT (*μ*M)	specific activity TBTKT^a^ (%)	MIC (*μ*g ml^−1^)/(*μ*M)
Oxythiamine	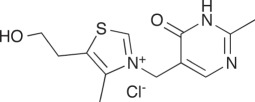	>30	130	>100/>332
5BPPO	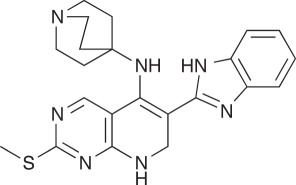	>30	100	>100/>227

^a^Specific activity of the TBTKT enzyme calculated compared with a 5% DMSO control taken as 100% activity.

## 5. Discussion

Here, we have successfully cloned, expressed and characterized, both kinetically and structurally, TBTKT, the first TKT enzyme to be characterized from mycobacteria. Kinetically, TBTKT has very similar kinetic constants to the TKT enzymes from yeast, *E. coli*, maize and spinach, despite the GDG to SDG mutation in the TKT consensus sequence. The TBTKT also displays broad substrate specificity for a range of phosphorylated sugars and in this respect differs from the mammalian TKT, as the human TKT enzyme has a much higher *K*_m,app_ and lower affinity for fructose-6-phosphate, compared with other identified substrates [11]. Phylogenetic studies have shown that mammalian TKT enzymes have diverged distinctly from equivalent TKTs in plants, yeast and bacteria, and therefore may have diversified to play a different, more selective role in humans.

Structurally, TBTKT consists of three domains with the same overall fold and domain topology as those of other TKTs whose structures have been determined [11,13,24,25,56,61,62]. Dimer formation and binding of the TPP cofactor occur via domains I and II, with domain III being more likely to play a regulatory role in substrate recognition as shown for the *E. coli* counterpart [28]. The recently reported structure of the human TKT structure [11] has allowed us to determine key structural differences between the human TKT enzyme and the TBTKT. Although the fold of the human TKT structure is similar overall to that of the TBTKT structure, despite only 27 per cent amino acid sequence similarity, we have identified important differences between the two structures, which may be exploited with a view to identifying specific inhibitors of TBTKT. It is important to ensure that inhibitors of the TKT enzyme in *M. tuberculosis* show specificity and do not inhibit human TKT or other thiamine-dependent enzymes such as pyruvate dehydrogenase, in order to minimize potential side-effects associated with a decrease in thiamine availability [22]. First, the substrate-binding channel in the human TKT enzyme is narrower than the TBTKT equivalent. This structural difference is believed to result in a more selective substrate specificity of human TKT compared with TBTKT [11]. The entrance of this substrate channel in the human TKT enzyme contains a lysine residue (Lys260) that is predicted to be involved in the binding of the phosphate of the sugar substrate [11]. This lysine residue is replaced by smaller residues in TBTKT and yeast TKT (Ala285 and Ala265, respectively), leaving more space to accommodate larger substrates (figure 5*b*).

TPP cofactor binding differs slightly between TBTKT and human TKT. The hydrophobic core that binds the pyrimidine ring of TPP in TBTKT is more hydrophilic in the human TKT enzyme. Moreover, the human enzyme displays a quasi-irreversible binding of TPP, which has not been observed for the yeast and *E. coli* homologues, in which the activity of recombinant human TKT in not dependent upon an excess of TPP or a divalent cation in the assay mixture. This is believed to be conferred in part by residue Gln189 (replaced by Ile211 in TBTKT) by sterically hindering cofactor dissociation [11] (figure 5*b*).

Another important difference between human and mycobacterial TKTs is the absence of the characteristic TKT five-histidine cluster from human TKT, as one of these histidines (His481 in yeast and His503 in TBTKT) has been replaced by a glutamine (Gln428; figure 5*b*). In yeast, His481 has mutated to Gln481, resulting in an increase in K_m,app_ values for X5P from 70 μM in wild-type yeast TKT to 4080 μM in the His481Gln yeast TKT mutant, suggesting a role for this residue in catalysis and substrate recognition in the activation of TPP through proton abstraction of the 4′-imino group of the cofactor [15].

Overall, the human TKT enzyme is 87 amino acids shorter than the TBTKT enzyme. The deletions in the human TKT correspond to two loops, between amino acids 153–167 and amino acids 413–432 in TBTKT. No possible interactions are predicted to occur between these loops and substrate or TPP. Also, the linker region between domains I and II (residues 300–376) is longer in TBTKT and forms a helix-turn-helix motif that may confer some specificity. On the basis of these structurally identified differences, it should be possible to design specific inhibitors rationally. Indeed, it is encouraging that oxythiamine and 5BPPO both inhibit human TKT [60], yet showed no inhibition of TBTKT activity or the growth *in vitro* of *M. tuberculosis*.

TBTKT represents a potential novel target for anti-tubercular therapy because it is predicted to be essential for *in vitro* growth of *M. tuberculosis* [4]. Additionally, TBTKT is believed to have an important role in production of precursors for the biosynthesis of the arabinans essential for the cell wall [31]. The *tkt* gene has been shown to be twofold upregulated 6 days after bacterial infection of host macrophages, and this probably reflects a response to the stress and toxic oxygen metabolites within this environment [10]. Additionally, this pathway is also responsible for the production of erythrose-6-phosphate that feeds into the shikamate pathway, producing folate, a *de novo* process in prokaryotic species. *Escherichia coli* mutants that lack TKT activity are auxotrophic for shikimic acid [63], while yeast mutants that are TKT-deficient are auxotrophic for aromatic amino acids [64]. TKT inhibitors are being considered for development for cancer therapy because TKT activity is upregulated in proliferative cells, and specificity for TKT enzymes in cancer cells can therefore be achieved through targeting an upregulated metabolic process [60].

In summary, TBTKT is the first TKT to have been characterized both structurally and biochemically from a mycobacterial species. The structure has revealed that, although the overall fold, domain organization and active-site architecture are very similar to those of other known TKTs, there are key structural and kinetic differences between the human TKT and TBTKT enzymes. This will enable us to exploit the TBTKT structure for rational drug design in an effort to find novel agents for antitubercular therapy.

## Supplementary Material

ESM_Figure_1

## References

[1] Dye C. 2006 Global epidemiology of tuberculosis. Lancet 367, 938–940 10.1016/S0140-6736(06)68384-0 (doi:10.1016/S0140-6736(06)68384-0) 16546542

[2] Kim DH 2008 Treatment outcomes and long-term survival in patients with extensively drug-resistant tuberculosis. Am. J. Respir. Crit. Care Med. 178, 1075–1082 10.1164/rccm.200801-132OC (doi:10.1164/rccm.200801-132OC) 18703792

[3] Cole ST 1998 Deciphering the biology of *Mycobacterium tuberculosis* from the complete genome sequence. Nature 393, 537–544 10.1038/31159 (doi:10.1038/31159) 9634230

[4] Sassetti CM Boyd DH Rubin EJ. 2003 Genes required for mycobacterial growth defined by high density mutagenesis. Mol. Microbiol. 48, 77–84 10.1046/j.1365-2958.2003.03425.x (doi:10.1046/j.1365-2958.2003.03425.x) 12657046

[5] Cole ST 2001 Massive gene decay in the leprosy bacillus. Nature 409, 1007–1011 10.1038/35059006 (doi:10.1038/35059006) 11234002

[6] Gu S Chen J Dobos KM Bradbury EM Belisle JT Chen X. 2003 Comprehensive proteomic profiling of the membrane constituents of a *Mycobacterium tuberculosis* strain. Mol. Cell Proteomics 2, 1284–1296 10.1074/mcp.M300060-MCP200 (doi:10.1074/mcp.M300060-MCP200) 14532352

[7] Malen H Berven FS Fladmark KE Wiker HG. 2007 Comprehensive analysis of exported proteins from *Mycobacterium tuberculosis* H37Rv. Proteomics 7, 1702–1718 10.1002/pmic.200600853 (doi:10.1002/pmic.200600853) 17443846

[8] Mawuenyega KG Forst CV Dobos KM Belisle JT Chen J Bradbury EM Bradbury AR Chen X. 2005 *Mycobacterium tuberculosis* functional network analysis by global subcellular protein profiling. Mol. Biol. Cell 16, 396–404 10.1091/mbc.E04-04-0329 (doi:10.1091/mbc.E04-04-0329) 15525680 PMC539182

[9] Rosenkrands I King A Weldingh K Moniatte M Moertz E Andersen P. 2000 Towards the proteome of *Mycobacterium tuberculosis*. Electrophoresis 21, 3740–3756 10.1002/1522-2683(200011)21:17<3740::AID-ELPS3740>3.0.CO;2-3 (doi:10.1002/1522-2683(200011)21:17<3740::AID-ELPS3740>3.0.CO;2-3) 11271494

[10] Triccas JA Berthet FX Pelicic V Gicquel B. 1999 Use of fluorescence induction and sucrose counterselection to identify *Mycobacterium tuberculosis* genes expressed within host cells. Microbiology 145, 2923–2930 10537214 10.1099/00221287-145-10-2923

[11] Mitschke L Parthier C Schroder-Tittmann K Coy J Ludtke S Tittmann K. 2010 The crystal structure of human transketolase and new insights into its mode of action. J. Biol. Chem. 285, 31 559–31 570 10.1074/jbc.M110.149955 (doi:10.1074/jbc.M110.149955) PMC2951230 20667822

[12] Schenk G Duggleby RG Nixon PF. 1998 Heterologous expression of human transketolase. Int. J. Biochem. Cell Biol. 30, 369–378 10.1016/S1357-2725(97)00154-4 (doi:10.1016/S1357-2725(97)00154-4) 9611778

[13] Lindqvist Y Schneider G Ermler U Sundstrom M. 1992 Three-dimensional structure of transketolase, a thiamine diphosphate dependent enzyme, at 2.5 A resolution. EMBO J. 11, 2373–2379 1628611 10.1002/j.1460-2075.1992.tb05301.x PMC556711

[14] Schneider G Lindqvist Y. 1998 Crystallography and mutagenesis of transketolase: mechanistic implications for enzymatic thiamin catalysis. Biochim. Biophys. Acta 1385, 387–398 10.1016/S0167-4838(98)00082-X (doi:10.1016/S0167-4838(98)00082-X) 9655943

[15] Wikner C Nilsson U Meshalkina L Udekwu C Lindqvist Y Schneider G. 1997 Identification of catalytically important residues in yeast transketolase. Biochemistry 36, 15 643–15 649 10.1021/bi971606b (doi:10.1021/bi971606b) 9398292

[16] Sprenger GA Schorken U Sprenger G Sahm H. 1995 Transketolase A of *Escherichia coli* K12. Purification and properties of the enzyme from recombinant strains. Eur. J. Biochem. 230, 525–532 10.1111/j.1432-1033.1995.0525h.x (doi:10.1111/j.1432-1033.1995.0525h.x) 7607225

[17] Gerhardt S 2003 Structure and properties of an engineered transketolase from maize. Plant Physiol. 132, 1941–1949 10.1104/pp.103.020982 (doi:10.1104/pp.103.020982) 12913150 PMC181279

[18] Flechner A Dressen U Westhoff P Henze K Schnarrenberger C Martin W. 1996 Molecular characterization of transketolase (EC 2.2.1.1) active in the Calvin cycle of spinach chloroplasts. Plant Mol. Biol. 32, 475–484 10.1007/BF00019099 (doi:10.1007/BF00019099) 8980496

[19] Joshi S Singh AR Kumar A Misra PC Siddiqi MI Saxena JK. 2008 Molecular cloning and characterization of *Plasmodium falciparum* transketolase. Mol. Biochem. Parasitol. 160, 32–41 10.1016/j.molbiopara.2008.03.005 (doi:10.1016/j.molbiopara.2008.03.005) 18456347

[20] Fiedler E Golbik R Schneider G Tittmann K Neef H Konig S Hubner G. 2001 Examination of donor substrate conversion in yeast transketolase. J. Biol. Chem. 276, 16 051–16 058 10.1074/jbc.M007936200 (doi:10.1074/jbc.M007936200) 11278369

[21] Kochetov GA. 1982 Transketolase from yeast, rat liver, and pig liver. Methods Enzymol. 90, 209–223 10.1016/S0076-6879(82)90128-8 (doi:10.1016/S0076-6879(82)90128-8) 6759853

[22] Schenk G Duggleby RG Nixon PF. 1998 Properties and functions of the thiamin diphosphate dependent enzyme transketolase. Int. J. Biochem. Cell Biol. 30, 1297–1318 10.1016/S1357-2725(98)00095-8 (doi:10.1016/S1357-2725(98)00095-8) 9924800

[23] Zhao G Winkler ME. 1994 An *Escherichia coli* K-12 tktA tktB mutant deficient in transketolase activity requires pyridoxine (vitamin B6) as well as the aromatic amino acids and vitamins for growth. J. Bacteriol. 176, 6134–6138 7928977 10.1128/jb.176.19.6134-6138.1994 PMC196835

[24] Nikkola M Lindqvist Y Schneider G. 1994 Refined structure of transketolase from *Saccharomyces cerevisiae* at 2.0 A resolution. J. Mol. Biol. 238, 387–404 10.1006/jmbi.1994.1299 (doi:10.1006/jmbi.1994.1299) 8176731

[25] Asztalos P Parthier C Golbik R Kleinschmidt M Hubner G. Weiss MS Friedemann R Wille G Tittmann K. 2007 Strain and near attack conformers in enzymic thiamin catalysis: X-ray crystallographic snapshots of bacterial transketolase in covalent complex with donor ketoses xylulose 5-phosphate and fructose 6-phosphate, and in noncovalent complex with acceptor aldose ribose 5-phosphate. Biochemistry 46, 12 037–12 052 10.1021/bi700844m (doi:10.1021/bi700844m) 17914867

[26] Veitch NJ Maugeri DA Cazzulo JJ Lindqvist Y Barrett MP. 2004 Transketolase from *Leishmania mexicana* has a dual subcellular localization. Biochem. J. 382, 759–767 10.1042/BJ20040459 (doi:10.1042/BJ20040459) 15149284 PMC1133835

[27] Littlechild J Turner N Hobbs G Lilly M Rawas A Watson H. 1995 Crystallization and preliminary X-ray crystallographic data with *Escherichia coli* transketolase. Acta Crystallogr. D Biol. Crystallogr. 51, 1074–1076 10.1107/S0907444995005415 (doi:10.1107/S0907444995005415) 15299777

[28] Costelloe SJ Ward JM Dalby PA. 2008 Evolutionary analysis of the TPP-dependent enzyme family. J. Mol. Evol. 66, 36–49 10.1007/s00239-007-9056-2 (doi:10.1007/s00239-007-9056-2) 18043855

[29] Usmanov RA Kochetov GA. 1983 Function of the arginine residue in the active center of baker's yeast transketolase. Biokhimiia 48, 772–781 6347264

[30] Kobori Y Myles DC Whitesides GM. 1992 Substrate specificity and carbohydrate synthesis using transketolase. J. Organ. Chem. 57, 5899–5907 10.1021/jo00048a023 (doi:10.1021/jo00048a023)

[31] Wolucka BA. 2008 Biosynthesis of D-arabinose in mycobacteria—a novel bacterial pathway with implications for antimycobacterial therapy. FEBS J. 275, 2691–2711 10.1111/j.1742-4658.2008.06395.x (doi:10.1111/j.1742-4658.2008.06395.x) 18422659

[32] Roos AK Andersson CE Bergfors T Jacobsson M Karlen A Unge T Jones TA Mowbray SL. 2004 *Mycobacterium tuberculosis* ribose-5-phosphate isomerase has a known fold, but a novel active site. J. Mol. Biol. 335, 799–809 10.1016/j.jmb.2003.11.021 (doi:10.1016/j.jmb.2003.11.021) 14687575

[33] Argyrou A Jin L Siconilfi-Baez L Angeletti RH Blanchard JS. 2006 Proteome-wide profiling of isoniazid targets in *Mycobacterium tuberculosis*. Biochemistry 45, 13 947–13 953 10.1021/bi061874m (doi:10.1021/bi061874m) PMC2519606 17115689

[34] Banerjee A Dubnau E Quemard A Balasubramanian V Um KS Wilson T Collins D de Lisle G Jacobs WR. Jr, 1994 inhA, a gene encoding a target for isoniazid and ethionamide in *Mycobacterium tuberculosis*. Science 263, 227–230 10.1126/science.8284673 (doi:10.1126/science.8284673) 8284673

[35] Mdluli K Slayden RA Zhu Y Ramaswamy S Pan X Mead D Crane DD Musser JM Barry CE. 3rd, 1998 Inhibition of a *Mycobacterium tuberculosis* beta-ketoacyl ACP synthase by isoniazid. Science 280, 1607–1610 10.1126/science.280.5369.1607 (doi:10.1126/science.280.5369.1607) 9616124

[36] Belanger AE Besra GS Ford ME Mikusova K Belisle JT Brennan PJ Inamine JM. 1996 The embAB genes of Mycobacterium avium encode an arabinosyl transferase involved in cell wall arabinan biosynthesis that is the target for the antimycobacterial drug ethambutol. Proc. Natl Acad. Sci. USA 93, 11 919–11 924 10.1073/pnas.93.21.11919 PMC38159 8876238

[37] Caceres NE Harris NB Wellehan JF Feng Z Kapur V Barletta RG. 1997 Overexpression of the D-alanine racemase gene confers resistance to D-cycloserine in *Mycobacterium smegmatis*. J. Bacteriol. 179, 5046–5055 9260945 10.1128/jb.179.16.5046-5055.1997 PMC179361

[38] David HL Takayama K Goldman DS. 1969 Susceptibility of mycobacterial D-alanyl-D-alanine synthetase to D-cycloserine. Am. Rev. Respir. Dis. 100, 579–581 4981706 10.1164/arrd.1969.100.4.579

[39] Makarov V 2009 Benzothiazinones kill *Mycobacterium tuberculosis* by blocking arabinan synthesis. Science 324, 801–804 10.1126/science.1171583 (doi:10.1126/science.1171583) 19299584 PMC3128490

[40] Kabsch W. 2010 Xds. Acta Crystallogr. D Biol. Crystallogr. 66, 125–132 10.1107/S0907444909047337 (doi:10.1107/S0907444909047337) 20124692 PMC2815665

[41] McCoy AJ Grosse-Kunstleve RW Adams PD Winn MD Storoni LC Read RJ. 2007 Phaser crystallographic software. J. Appl. Crystallogr. 40, 658–674 10.1107/S0021889807021206 (doi:10.1107/S0021889807021206) 19461840 PMC2483472

[42] Arnold K Bordoli L Kopp J Schwede T. 2006 The SWISS-MODEL workspace: a web-based environment for protein structure homology modelling. Bioinformatics 22, 195–201 10.1093/bioinformatics/bti770 (doi:10.1093/bioinformatics/bti770) 16301204

[43] Jones TA Zou JY Cowan SW Kjeldgaard M. 1991 Improved methods for building protein models in electron density maps and the location of errors in these models. Acta Crystallogr. A 47, 110–119 10.1107/S0108767390010224 (doi:10.1107/S0108767390010224) 2025413

[44] Emsley P Lohkamp B Scott WG Cowtan K. 2010 Features and development of Coot. Acta Crystallogr. D Biol. Crystallogr. 66, 486–501 10.1107/S0907444910007493 (doi:10.1107/S0907444910007493) 20383002 PMC2852313

[45] Murshudov GN Vagin AA Dodson EJ. 1997 Refinement of macromolecular structures by the maximum-likelihood method. Acta Crystallogr. D Biol. Crystallogr. 53, 240–255 10.1107/S0907444996012255 (doi:10.1107/S0907444996012255) 15299926

[46] Diederichs K Karplus PA. 1997 Improved R-factors for diffraction data analysis in macromolecular crystallography. Nat. Struct. Biol. 4, 269–275 10.1038/nsb0497-269 (doi:10.1038/nsb0497-269) 9095194

[47] Chenna R Sugawara H Koike T Lopez R Gibson TJ Higgins DG Thompson JD. 2003 Multiple sequence alignment with the Clustal series of programs. Nucleic Acids Res. 31, 3497–3500 10.1093/nar/gkg500 (doi:10.1093/nar/gkg500) 12824352 PMC168907

[48] Gouet P Courcelle E Stuart DI Metoz F. 1999 ESPript: analysis of multiple sequence alignments in PostScript. Bioinformatics 15, 305–308 10.1093/bioinformatic/15.4.305 (doi:10.1093/bioinformatic/15.4.305) 10320398

[49] Kochetov GA. 1982 Determination of transketolase activity via ferricyanide reduction. Methods Enzymol. 89, 43–44 10.1016/S0076-6879(82)89009-5 (doi:10.1016/S0076-6879(82)89009-5) 7144583

[50] Schenk G Layfield R Candy JM Duggleby RG Nixon PF. 1997 Molecular evolutionary analysis of the thiamine-diphosphate-dependent enzyme, transketolase. J. Mol. Evol. 44, 552–572 10.1007/PL00006179 (doi:10.1007/PL00006179) 9115179

[51] Matthews BW. 1968 Solvent content of protein crystals. J. Mol. Biol. 33, 491–497 10.1016/0022-2836(68)90205-2 (doi:10.1016/0022-2836(68)90205-2) 5700707

[52] Holm L Rosenstrom P. 2010 Dali server: conservation mapping in 3D. Nucleic Acids Res. 38, W545–W549 10.1093/nar/gkq366 (doi:10.1093/nar/gkq366) 20457744 PMC2896194

[53] Teige M Melzer M Suss KH. 1998 Purification, properties and in situ localization of the amphibolic enzymes D-ribulose 5-phosphate 3-epimerase and transketolase from spinach chloroplasts. Eur. J. Biochem. 252, 237–244 10.1046/j.1432-1327.1998.2520237.x (doi:10.1046/j.1432-1327.1998.2520237.x) 9523694

[54] Fiedler E Thorell S Sandalova T Golbik R Konig S Schneider G. 2002 Snapshot of a key intermediate in enzymatic thiamin catalysis: crystal structure of the alpha-carbanion of (alpha,beta-dihydroxyethyl)-thiamin diphosphate in the active site of transketolase from *Saccharomyces cerevisiae*. Proc. Natl Acad. Sci. USA 99, 591–595 10.1073/pnas.022510999 (doi:10.1073/pnas.022510999) 11773632 PMC117350

[55] Kremer AB Egan RM Sable HZ. 1980 The active site of transketolase. Two arginine residues are essential for activity. J. Biol. Chem. 255, 2405–2410 7358678

[56] Nilsson U Meshalkina L Lindqvist Y Schneider G. 1997 Examination of substrate binding in thiamin diphosphate-dependent transketolase by protein crystallography and site-directed mutagenesis. J. Biol. Chem. 272, 1864–1869 10.1074/jbc.272.3.1864 (doi:10.1074/jbc.272.3.1864) 8999873

[57] Breslow R. 1958 On the mechanism of thiamine action. IV. Evidence from studies on model systems. J. Am. Chem. Soc. 80, 3719–3726 10.1021/ja01547a064 (doi:10.1021/ja01547a064)

[58] Krampitz LO. 1969 Catalytic functions of thiamin diphosphate. Annu. Rev. Biochem. 38, 213–240 10.1146/annurev.bi.38.070169.001241 (doi:10.1146/annurev.bi.38.070169.001241) 4896238

[59] Rais B 1999 Oxythiamine and dehydroepiandrosterone induce a G1 phase cycle arrest in Ehrlich's tumor cells through inhibition of the pentose cycle. FEBS Lett. 456, 113–118 10.1016/S0014-5793(99)00924-2 (doi:10.1016/S0014-5793(99)00924-2) 10452541

[60] Du MX Sim J Fang L Yin Z Koh S Stratton J Pons J Wang JJ Carte B. 2004 Identification of novel small-molecule inhibitors for human transketolase by high-throughput screening with fluorescent intensity (FLINT) assay. J. Biomol. Screen 9, 427–433 10.1177/1087057104263913 (doi:10.1177/1087057104263913) 15296642

[61] Wikner C Meshalkina L Nilsson U Backstrom S Lindqvist Y Schneider G. 1995 His103 in yeast transketolase is required for substrate recognition and catalysis. Eur. J. Biochem. 233, 750–755 10.1111/j.1432-1033.1995.750_3.x (doi:10.1111/j.1432-1033.1995.750_3.x) 8521838

[62] Nilsson U Lindqvist Y Kluger R Schneider G. 1993 Crystal structure of transketolase in complex with thiamine thiazolone diphosphate, an analogue of the reaction intermediate, at 2.3 A resolution. FEBS Lett. 326, 145–148 10.1016/0014-5793(93)81779-Y (doi:10.1016/0014-5793(93)81779-Y) 8325361

[63] Josephson BL Fraenkel DG. 1969 Transketolase mutants of *Escherichia coli*. J. Bacteriol. 100, 1289–1295 4902809 10.1128/jb.100.3.1289-1295.1969 PMC250317

[64] Sundstrom M Lindqvist Y Schneider G Hellman U Ronne H. 1993 Yeast TKL1 gene encodes a transketolase that is required for efficient glycolysis and biosynthesis of aromatic amino acids. J. Biol. Chem. 268, 24 346–24 352 8226984

