# Supplementary material for: Structure and function of the transketolase from Mycobacterium tuberculosis and comparison with the human enzyme
Source: Open Biol. 2012 Jan;2(1):110026. doi: 10.1098/rsob.110026 (PMC3352088; doi:10.1098/rsob.110026)
Supplement: ESM_Figure_1 [file rsob110026-s1.pdf]

Supplementary Information

Figure S1: Sequence comparison of TBTKT and homologues. Sequence alignment of TBTKT versus TKTs from *M. bovis* BCG, *M. leprae, M. marinum*, *M. smegmatis*, *Corynebacterium glutamicum, E. coli, Bacillus anthracis, Bacillus subtilus, Staphyloccocus auerus,* maize, spinach, yeast, mouse, rat and human using the programs ClustalW2 and ESPript 2.2 (46,47). Numbering corresponds to the sequence of TBTKT. Identical residues are indicated by a red background, and conserved residues are indicated by red characters.

```
                                                 1       10        20
Mtuberculosis    .........................................MTTLEEIS...ALTRPRHPDYWTEIDSA.
MbovisBCG        .........................................MTTLEEIS...ALTRPRHPDDWTEIDSA.
Mleprae          .........................................MTTLDQIS...TLTQPRHPDDWTEIDSA.
Mmarinum         .........................................MTTLEEIS...ALTQPRHPDDWTEIDSA.
Msmegmatis       .........................................MTTAEEIT...ALTQPNHPDDWTDLDTL.
Cglutamicum      .........................................MTTLTLSPELQALTVRNYPSDWSDVDTK.
Ecoli            ...........................................................MSSRKELANA.
Banthracis       ..........................................MSSGVDLGTENLYFQSNAMSHSIEQL..
Bsubtilis        .........................................................MDTIEKK......
Staphylococcus   ..........................................................MFNEKDQL....
Maize            .......................................................GAVETLQGKAAT...
Spinach          MAASSSLSTLSHHQTLLSHPKTHLPTTPASSLLVPTTSSKVNGVLLKSTSSSRRLRVGSASAVVRAAAVE
Yeast            .......................................................MTQFTDIDKLA....
Mouse            ..........................................................MEGYHKPDQQKL
Rat              ..........................................................MEGYHKPDQQKL
Human            ..........................................................MESYHKPDQQKL
```

```
                              30        40        50        60        70
Mtuberculosis    .............AVDTIRVLAAD...AVQKVGNGHPGTAMSLAPLAYTLFQRTMRHDPSDTHWLGRDRF
MbovisBCG        .............AVDTIRVLAAD...AVQKVGNGHPGTAMSLAPLAYTLFQRTMRHDPSDTHWLGRDRF
Mleprae          .............AVDTIRVLAAD...AVQKAGNGHPGTAMSLAPLAYTLFQRTLRHDPNDTAWLGRDRF
Mmarinum         .............AVDTARVLAAD...AVQKVGNGHPGTAMSLAPLAYTLFQRAMRHDPSDTTWLGRDRF
Msmegmatis       .............SVDTARVLAAD...AVQKVGNGHPGTAMSLAPLAYTLFQRQMRHDPSDTHWLGRDRF
Cglutamicum      .............AVDTVRVLAAD...AVENCGSGHPGTAMSLAPLAYTLYQRVMNVDPQDTNWAGRDRF
Ecoli            .............IRALSM....D...AVQKAKSGHPGAPMGMADIAEVLWRDFLKHNPQNPSWADRDRF
Banthracis       .............SINTIRTLSID...AIEKANSGHPGMPMGAAPMAYTLWTQFMKHNPNNPTWFNRDRF
Bsubtilis        .............SVATIRTLSID...AIEKANSGHPGMPMGAAPMAYTLWTKFMNVSPANPGWFNRDRF
Staphylococcus   .............AVDTLRALSID...TIEKANSGHPGLPMGAAPMAYTLWTRHLNFNPQSKDYFNRDRF
Maize            .......GELLEKSVNTIRFLAID...AVEKANSGHPGLPMGCAPMGHVLYDEVMRYNPKNPYWFNRDRF
Spinach          ALESTDIDQLVEKSVNTIRFLAID...AVEKANSGHPGLPMGCAPMGHILYDEIMRYNPKNPYWFNRDRF
Yeast            ..............VSTIRILAVD...TVSKANSGHPGAPLGMAPAAHVLWSQ.MRMNPTNPDWINRDRF
Mouse            QAL...........KDTANRLRISSIQATTAAGSGHPTSCCSAAEIMAVLFFHTMRYKALDPRNPHNDRF
Rat              QAL...........KDTANRLRISSIQATTAAGSGHPTSCCSAAEIMAVLFFHTMRYKALDPRNPHNDRF
Human            QAL...........KDTANRLRISSIQATTAAGSGHPTSCCSAAEIMAVLFFHTMRYKSQDPRNPHNDRF
```

```
                 80        90        100          110       120       130       140
Mtuberculosis    VLSAGHSSLTLYIQLYLGGFGLE..LSDIESLRTWGSKTPGHPEFRHTPGVEITTGPLGQGLASAVGMAM
MbovisBCG        VLSAGHSSLTLYIQLYLGGFGLE..LSDIESLRTWGSKTPGHPEFRHTPGVEITTGPLGQGLASAVGMAM
Mleprae          VLSAGHSSLTLYIQLYLGGFGLE..LSDIESLRTWGSTTPGHPEFRHTKGVEITTGPLGQGLASAVGMAM
Mmarinum         VLSCGHSSLTLYIQLYLGGFGLE..LSDIESLRTWGSKTPGHPEFRHTAGVEITTGPLGQGLASAVGMAM
Msmegmatis       ILSCGHSSLTLYIQLYLGGFGLE..LSDIESLRTWGSKTPGHPEFRHTKGVEITTGPLGQGLASAVGMAM
Cglutamicum      VLSCGHSSLTQYIQLYLGGFGLE..MDDLKALRTWDSLTPGHPEYRHTKGVEITTGPLGQGLASAVGMAM
Ecoli            VLSNGHGSMLIYSLLHLTGYDLP..MEELKNFRQLHSKTPGHPEVGYTAGVETTTGPLGQGIANAVGMAI
Banthracis       VLSAGHGSMLLYSLLHLSGYDVT..MDDLKNFRQWGSKTPGHPEYGHTAGVDATTGPLGQGIATAVGMAM
Bsubtilis        VLSAGHGSALLYSMLHLSGFDLS..IEDLKGFRQWGSKTPGHPEFGHTAGVDATTGPLGQGIAMAVGMAI
Staphylococcus   VLSAGHGSALLYSLLH.VSGSLE..LEELKQFRQWGSKTPGHPEYRHTDGVEVTTGPLGQGFAMSVGLAL
Maize            VLSAGHGCMLQYALLHLAGYDSV.KEEDLKQFRQWGSRTPGHPENFETPGVEVTTGPLGQGIANAVGLAL
Spinach          VLSAGHGCMLQYALLHLAGYDSV.LEEDLKTFRQWGSRIPGHPENFETPGVEVTTGPLGQGIANAVGLAL
Yeast            VLSNGHAVALLYS..MLHLTGYDLSIEDLKQFRQLGSRTPGHPEFELP.GVEVTTGPLGQGISNAVGMAM
Mouse            VLSKGHAAPILYAVWAEAGFLPEAELLNLRKISSDLDGHPVPKQ.AFTDVA...TGSLGQGLGAACGMAY
Rat              VLSKGHAAPILYAVWAEAGFLPEAELLNLRKISSDLDGHPVPKQ.AFTDVA...TGSLGQGLGAACGMAY
Human            VLSKGHAAPILYAVWAEAGFLAEAELLNLRKISSDLDGHPVPKQ.AFTDVA...TGSLGQGLGAACGMAY
```

```
                 150       160       170       180       190       200       210
Mtuberculosis    ASRYERGLFDPDAEPGASPFDHYIYVIASDGDIEEGVTSEASSLAAVQQLGNLIVFYDRNQISIED.DTN
MbovisBCG        ASRYERGLFDPDAEPGASPFDHYIYVIASDGDIEEGVTSEASSLAAVQQLGNLIVFYDRNQISIED.DTN
Mleprae          ASRYERGLFDPDAEPGASPFDHYIYVIASDGDIEEGVTSEASSLAAVQQLGNLIVFYDHNQISIEG.DTK
Mmarinum         AARYERGLFDPDAEPGTSPFDHHIYVIASDGDIEEGVTSEASSLAAVQQLGNLIVFYDHNKISIED.DTN
Msmegmatis       AARYERGLFDPDAAPGTSPFDHYIYVVASDGDMEEGVTSEASSLAGTQQLGNLIVIWDDNKISIEH.NTD
Cglutamicum      AARRERGLFDPTAAEGESPFDHHIYVIASDGDLQEGVTSEASSIAGTQQLGNLIVFWDDNRISIED.NTE
Ecoli            AEKTLA...AQFNRPGHDIVDHYTYAFMGDGCMMEGISHEVCSLAGTLKLGKLIAFYDDNGISIDG.HVE
Banthracis       AERHLA...AKYNRDAYNIVDHYTYAICGDGDLMEGVSAEASSLAAHLQLGRLVVLYDSNDISLDG.DLN
Bsubtilis        AERHLA...ETYNRDSFNVVDHYTYSICGDGDLMEGISSEAASLAGHLQLGRLIVLYDSNDISLDG.DLD
Staphylococcus   AEDHLA...GKFNKEGYNVVDHYTYVLASDGDLMEGISHEAASFAGHNKLSKLVVLYDSNDISLDG.ELN
Maize            AEKHLA...ARFNKPDSEIVDHYTYVILGDGCQMEGIANEACSLAGHWGLGKLIAFYDDNHISIDG.DTE
Spinach          AEKHLA...ARFNKPDAEIVDHYTYVILGDGCQMEGIAQEACSLAGHWGLGKLIAFYDDNHISIDG.DTA
Yeast            AQANLA...ATYNKPGFTLSDNYTYVFLGDGCLQEGISSEASSLAGHLKLGNLIAIYDDNKITIDG.ATS
Mouse            TGKYFD...........KASYRVYCML.GDGEVSEGSVWEAMAFAGIYKLDNLVAIFDINRLGQSDPAPL
Rat              TGKYFD...........KASYRVYCML.GDGEVSEGSVWEAMAFAGIYKLDNLVAIFDINRLGQSDPAPL
Human            TGKYFD...........KASYRVYCLL.GDGELSEGSVWEAMAFASIYKLDNLVAILDINRLGQSDPAPL
```

```
                   220       230       240       250       260       270
Mtuberculosis    IALCEDTAARYRAYGWHVQ.EVEGGE....NVVGIEEAIANAQAVTDRPSFIALRTVIGYPAPNLMDT..
MbovisBCG        IALCEDTAARYRAYGWHVQ.EVEGGE....NVVGIEEAIANAQAVTDRPSFIALRTVIGYPAPNLMDT..
Mleprae          ITLCEDTAARYRAYGWHVQ.EVEGGE....NVVGIEEAIANAKAATDRPSFISLRTIIGYPAPTLINT..
Mmarinum         IALCEDTAARYRAYGWHVQ.EVEGGE....NVTGIEEAIANAKAVTDRPSFISLRTIIGYPAPNMMNT..
Msmegmatis       IAFSEDVVARYRAYGWHVQ.EVQGGE....NVVGIEQALEEARKVTDKPSFIALRTIIGYPAPNKMNT..
Cglutamicum      IAFNEDVVARYKAYGWQTI.EVEAGE....DVAAIEAAVAEAKKDTKRPTFIRVRTIIGFPAPTMMNT..
Ecoli            GWFTDDTAMRFEAYGWHVI.RDIDGH....DAASIKRAVEEARAVTDKPSLLMCKTIIGFGSPNKAGT..
Banthracis       RSFSESVEDRYKAYGWQVI.RVEDGN....DIEAIAKAIEEAKADEKRPTLIEVRTTIGFGSPNKSGK..
Bsubtilus        RSFSENVKQRFEAMNWEVL.YVEDGN....NIEELTAAIEKARQNEKKPTLIEVKTTIGFGSPNRAGT..
Staphylococcus   KAFSENTKARFEAYGWNYL.LVKDGN....DLEEIDKAITTAK.SQEGPTIIEVKTTIGFGSPNKAGT..
Maize            IAFTEDVSTRFEALGWHTI.WVKNGNT...GYDDIRAAIKEAKAVTDKPTLIKVTTTIGFGSPNKANS..
Spinach          IAFTESVDLRFEALGWHVI.WVKNGNT...GYDEIRAAIKEAKTVTDKPTLIKVTTTIGFGSPNKSNS..
Yeast            ISFDEDVAKRYEAYGWEVL.YVENGNE...DLAGIAKAIAQAKLSKDKPTLIKMTTTIGYGSLHAGSH..
Mouse            QHQVDIYQKRCEAFGWHTIIVDGHSVEELCKAFGQAKHQPTAIIAKTFK...GRGITGIEDKEAWHGKPL
Rat              QHQVDVYQKRCEAFGWHAIIVDGHSVEELCKAFGQAKHQPTAIIAKTFK...GRGITGIEDKEAWHGKPL
Human            QHQMDIYQKRCEAFGWHAIIVDGHSVEELCKAFGQAKHQPTAIIAKTFK...GRGITGVEDKESWHGKPL

                 280         290       300       310       320          330
Mtuberculosis    .GKA........HGAALGDDEVAAVKKIVGFDPDKTFQVREDVLTHTRGLVA....RGKQAHERWQLEFD
MbovisBCG        .GKA........HGAALGDDEVAAVKKIVGFDPDKTFQVREDVLTHTRGLVA....RGKQAHERWQLEFD
Mleprae          .GKA........HGAALGDEDVAATKRILGFDPDKTFAVREDVITHTRGLIA....RGKEAHERWQLEFE
Mmarinum         .GKV........HGAALGDEEVAAVKTVLGFDPNNAFEVREDVITHTRGLVA....RGKEAHESWQADFE
Msmegmatis       .GGV........HGSALGADEVAATKKILGFDPEKNFEVRDEVIAHTRELVA....RGKKAHDEWQVSFD
Cglutamicum      .GAV........HGAALGAAEVAATKTELGFDPEAHFAIDDEVIAHTRSLAE....RAAQKKAAWQVKFD
Ecoli            .HDS........HGAPLGDAEIALTREQLGWKYAPFEIPSEIYAQWDAKEAGQAKESAWNEKFAAYAKAY
Banthracis       .SAS........HGSPLGVEETKLTKEAYAWTAEQDFHVAEEVYENFRKTVQD...VGETAQAEWNTMLG
Bsubtilus        .SGV........HGAPLGKEESKLTKEAYAWTYEEDFYVPSEVYEHFAVAVKE...SGEKKEQEWNAQFA
Staphylococcus   .NGV........HGAPLGEVERKLTFENYGLDPEKRFNVSEEVYEIFQNTMLK...RANEDESQWNSLLE
Maize            .YSV........HGSALGAKEVEATRQNLGWPYDTFFVPEDVKSHWSRHTPEG...AALEADWNAKFAEY
Spinach          .YSV........HGSALGSKEVEATRQNLGWPYEPFHVPEEVKKHWSRHTPEG...ASLEAEWNTKFAEY
Yeast            .SVAGAPLKAD.DVKQLKSKFGFNPDKSFVVPQE........VYDHYQKTILK...PGVEANNKWNKLFS
Mouse            PKNMAEQIIQEIYSQVQSKKKILATPPQEDAPSVDIANIRMPTPPSYKV.......GDKIATRKAYGLAL
Rat              PKNMAEQIIQEIYSQVQSKKKILATPPQEDAPSVDIANIRMPTPPNYKV.......GDKIATRKAYGLAL
Human            PKNMAEQIIQEIYSQIQSKKKILATPPQEDAPSVDIANIRMPSLPSYKV.......GDKIATRKAYGQAL

                 340        350       360       370        380       390       400
Mtuberculosis    AWARREPE.RKALLDRLLAQKLPDGWDADLPHWEPGSKAL.ATRAASGAVLSALGPKLPELWGGSADLAG
MbovisBCG        AWARREPE.RKALLDRLLAQKLPDGWDADLPHWEPGSKAL.ATRAASGAVLSALGPKLPELWGGSADLAG
Mleprae          AWAQREPE.RKALLDRLLAQQLPDGWDADLPNWEPRSKEL.ATRAASGAVLSAIGPKLPELWGGSADLAG
Mmarinum         AWAQREPQ.RKALLDRLTAQQLPDGWDADMPRWEPGSKAL.ATRAASGQVLAAIGPKLPELWGGSADLAG
Msmegmatis       EWAEREPE.RKELLDRLLAGKLPEGWDADLPHWDLDAKPV.ATRAASGAVLSAVGPKLPELWGGSADLAG
Cglutamicum      EWAAANPE.NKALFDRLNSRELPAGYADELPTWDADEKGV.ATRKASEAALQALGKTLPELWGGSADLAG
Ecoli            PQEAAEFT.RR...MKGEMPSDFDAKAKEFIAKLQANPAKIASRKASQNAIEAFGPLLPEFLGGSADLAP
Banthracis       EYAQAYPE.LANELQAAMNGLLPEGWEQNLPTYELGSKA..ATRNSSGAVINAIAESVPSFFGGSADLAG
Bsubtilus        KYKEVYPE.LAEQLELAIKGELPKDWDQEVPVYEKGSSL..ASRASSGEVLNGLAKKIPFFVGGSADLAG
Staphylococcus   KYAETYPE.LAEEFKLAISGKLPKNYKDELPRFELGHNG..ASRADSGTVIQAISKTVPSFFGGSADLAG
Maize            EKKYAD...DAATLKSIITGELPTGWVDALPKYTPESPGD.ATRNLSQQCL.NALA......NVVPGLIG
Spinach          EKKYPE...DATEFKSITTGEFPAGWEKALPTYTPETPGD.ATRNLSQQCL.NALA......KVIPGLLG
Yeast            EYQKKFPELGAELARRLS.GQLPANWESKLPTYTAKDSAV.ATRKLSETVLEDVYNQLPELIGGSADLTP
Mouse            AKLGHA...SDRIIAL..DGDTKNSTFSELFKKEHPDRFIECYIAEQ................NMVSIAV
Rat              AKLGHA...SDRIIAL..DGDTKNSTFSELFKKEHPDRFIECYIAEQ................NMVSIAV
Human            AKLGHA...SDRIIAL..DGDTKNSTFSEIFKKEHPDRFIECYIAEQ................NMVSIAV

                    410       420       430       440       450        460       470
Mtuberculosis    SNNTTIKGADSFGPPSISTKEYTAHWYGRTLHFGVREHAMGAILSGIVLHG.PTRAYGGTFLQFSDYMRP
MbovisBCG        SNNTTIKGADSFGPPSISTKEYTAHWYGRTLHFGVREHAMGAILSGIVLHG.PTRAYGGTFLQFSDYMRP
Mleprae          SNNTTIKDVDSFGPPSISTDEYTAHWYGRTLHFGVREHAMGAILSGIVLHG.PTRAYGGTFLQFSDYMRP
Mmarinum         SNNTTIKGADSFGPPAISTDDYTAHWYGRTLHFGVREHAMGAILSGIALHG.PTRAYGGTFLQFSDYMRP
Msmegmatis       SNNTTIKGANSFGPPSISTEDWNATWYGRTLHFGVREHAMGAILSGIVLHG.PTRAYGGTFLQFSDYMRP
Cglutamicum      SNNTVIKGSPSFGPESISTETWSAEPYGRNLHFGIREHAMGSILNGISLHG.GTRPYGGTFLIFSDYMRP
Ecoli            SNLTLWSG.........SKAINEDAA.GNYIHYGVREFGMTAIANGISLHG.GFLPYTSTFLMFVEYARN
Banthracis       SNKTYMNNEKD......FTRDDYS...GKNIWYGVREFAMGAAMNGIALHG.GLKTYGGTFFVFSDYLRP
Bsubtilus        SNKTTIKNAGD......FTAVDYS...GKNFWFGVREFAMGAALNGMALHG.GLRVFGGTFFVFSDYLRP
Staphylococcus   SNK...SNVND......ATDYSSETPEGKNVWFGVREFAMGAAVNGMAAHG.GLHPYGATFFVFSDYLKP
Maize            GSADLASSNMTLLKMFGDFQKDTAEE..RNVRFGVREHGMGAICNGIALHSPGFVPYCATFFVFTDYMRG
Spinach          GSADLASSNMTLLKMFGDFRRTHRKK..ETFRFGVREHGMGAICNGICLHSPGFVPYCATFFVFTDYMRG
Yeast            SNLTRWKEALDFQPPSSGSGNYSGR....YIRYGIREHAMGAIMNGISAFGANYKPYGGTFLNFVSYAAG
Mouse            GCAT............RDRTVPF.....CSTFAAFFTRAFDQIRMAAISESNINLCGSH...CGVSIGE
Rat              GCAT............RDRTVPF.....CSTFAAFFTRAFDQIRMAAISESNINLCGSH...CGVSIGE
Human            GCAT............RNRTVPF.....CSTFAAFFTRAFDQIRMAAISESNINLCGSH...CGVSIGE
```

```
                          480       490       500       510       520       530       540
Mtuberculosis    AV.RLAALMDIDTIYVWTHDSIGLGEDGPTHQPIEHLSALRAIPRLSVVRPADANETAYAWRTILARR..
MbovisBCG        AV.RLAALMDIDTIYVWTHDSIGLGEDGPTHQPIEHLSALRAIPRLSVVRPADANETAYAWRTILARR..
Mleprae          SV.RLASLMDIDTIYVWTHDSVGLGEDGPTHQPIEHLAALRAIPRLSVVRPADANETAYAWRTILARG..
Mmarinum         AV.RLAALMDIDPIYVWTHDSIGLGEDGPTHQPIEHLSALRAIPRLAVVRPADANETAYAWSTILARG..
Msmegmatis       AV.RLASLMDIDPIYVWTHDSIGLGEDGPTHQPIEHLAALRAIPNLSVVRPGDPNETAYAWKSVLERG..
Cglutamicum      AV.RLAALMETDAYYVWTHDSIGLGEDGPTHQPVETLAALRAIPGLSVLRPADANETAQAW....AAALE
Ecoli            AV.RMAALMKQRQVMVYTHDSIGLGEDGPTHQPVEQVASLRVTPNMSTWRPCDQVESAVAWKY.......
Banthracis       AI.RLAALMQLPVTYVFTHDSIAVGEDGPTHEPIEQLAALRAMPNVSVIRPADGNESVAAWRL.......
Bsubtilus        AI.RLAALMGLPVTYVFTHDSIAVGEDGPTHEPVEQLASLRAMPNLSLIRPADGNETAAAWKL.......
Staphylococcus   AL.RLSSIMGLNATFIFTHDSIAVGEDGPTHEPIEQLAGLRAIPNMNVIRPADGNETRVAWEV.......
Maize            AM.RISALSEAGVIYVMTHDSIGLGEDGPTHQPIEHLVSFRAMPNILMLRPADGNETAGAYKVA......
Spinach          AM.RISALSEAGVIYVMTHDSIGLGEDGPTHQPIEALSKFPAMPNILMLRPADGNETAGSYKVA......
Yeast            AV.RLSALSGHPVIWVATHDSIGVGEDGPTHQPIETLAHFRSLPNIQVWRPADGNE.VSAAYKN......
Mouse            DGPSQMAL........EDLAMFRSVPMS.TVF...YPSDGVATEKAVELAANTKGICFIRTSRPEN....
Rat              DGPSQMAL........EDLAMFRSVPMS.TVF...YPSDGVATEKAVELAANTKGICFIRTSRPEN....
Human            DGPSQMAL........EDLAMFRSVPTS.TVF...YPSDGVATEKAVELAANTKGICFIRTSRPEN....

                            550       560       570       580       590       600
Mtuberculosis    NGSGPVGLILTRQGVPVLD...GTD...AEGVARGGY...VLSDAGGLQPGEEPDVILIATGSEVQLAVA
MbovisBCG        NGSGPVGLILTRQGVPVLD...GTD...AEGVARGGY...VLSDAGGLQPGEEPDVILIATGSEVQLAVA
Mleprae          ANSGPVGLILTRQSVPVLE...GTN...TEGVARGGY...VLGDGGSSEAKEP.DVILIATGSEVQLAVA
Mmarinum         ATSGPVGLILTRQGVPVLA...GTN...AEGVARGGY...VLSDGADGDSSEDPDVVLIATGSEVQLAVE
Msmegmatis       SSSGPVGMILTRQPIPVLE...GTD...PEGVARGGY...VLGGTPEKNPD....VVIIGTGSELQLAVG
Cglutamicum      YKEGPKGLALTRQNVPVLE...GTKEKAAEGVRRGGY...VLVEGSKETPDVILMGS....GSEVQLAVN
Ecoli            GVERQDGPTALILSRQNLAQQERTEEQLA.NIARGGY...VLKDCAGQPELIF.....IATGSEVELAVA
Banthracis       ALESTNKPTALVLTRQDLPTLEGAKDDTYEKVAKGAY...VVSASKKETADVILL....ATGSEVSLAVE
Bsubtilus        AVQSTDHPTALVLTRQNLPTIDQTSEEALAGVEKGAY...VVSKSKNETPDALLI....ASGSEVGLAIE
Staphylococcus   ALESESTPTSLVLTRQNLP..VLDVPED.VVEEGVRK..GAYTVYGSEETPEFLLL..ASGSEVSLAVE
Maize            VL.NRKRPSILALSRQKLPHLPGTSI...EGVEKGGYTISDNSTGNKPDLIVM......GTGSELEIAAK
Spinach          VE.NRKTPSILALSRKKLPNLPGTSI...EGVEKGGYTITDNSSGNKPDVILI......GTGSELEIAAK
Yeast            SLESKHTPSIIALSRQNLPQLEGSSI...ESASKGGY...VLQDVANPDIILV......ATGSEVSLSVE
Mouse            AIIYSNNEDFQVGQAKV.....VLK.....SKDDQVT...VIGAGVTLHEALAAAE...SLKKDKI..SI
Rat              AIIYSNNEDFQVGQAKV.....VLK.....SKDDQVT...VIGAGVTLHEALAAAE...MLKKEKI..GV
Human            AIIYNNNEDFQVGQAKV.....VLK.....SKDDQVT...VIGAGVTLHEALAAAE...LLKKEKI..NI

                           610       620       630       640       650       660       670
Mtuberculosis    AQTLLADNDILARVVSMPCLEWFEAQPYEYRDAVLPPTVSARVAVEAGVAQCWHQLVGDTGEIV.SIEHY
MbovisBCG        AQTLLADNDILARVVSMPCLEWFEAQPYEYRDAVLPPTVSARVAVEAGVAQCWHQLVGDTGEIV.SIEHY
Mleprae          AQKLLADKDIIVRVVSMPCVEWFESQPYEYRDSVLPPSVSARVAVEAGVAQCWHKLVGDTGKIV.SIEHY
Mmarinum         ARKLLADNDIIARVVSMPCVEWFESQPEEYRDSVLPPSVSARVAVEAGIAQCWHKLVGDTGKIV.SIEHY
Msmegmatis       AQKILADKGITASVVSMPCVEWFESQPEEYRDSVLPPAVSARVAVEAAVAQSWYKLVGDTGEIV.SIEHY
Cglutamicum      AAKALEAEGVAARVVSVPCMDWFQEQDAEYIESVLPAAVTARVSVEAGIAMPWYRFLGTQGRAV.SLEHF
Ecoli            AYEKLTAEGVKARVVSMPSTDAFDKQDAAYRESVLPKAVTARVAVEAGIADYWYKYVGLNGAIV.GMTTF
Banthracis       AQKALAVDGVDASVVSMPSMDRFEAQTAEYKESVLPKAVTKRFAIEMGATFGWHRYVGLEGDVL.GIDTF
Bsubtilus        AQAELAKENIDVSVVSMPSMDRFEKQSDEYKNEVLPADVKKRLAIEMGSSFGWGKYTGLEGDVL.GIDRF
Staphylococcus   AAKDLEKQGKSVRVVSMPNWNAFEQQSEEYKESVIPSSVTKRVAIEMASPLGWHKYVGTAGKVI.AIDGF
Maize            AADELRKEGKTVRVVSFVSWELFDEQSDEYKESVLPAAVTARISIEAGSTLGWQKYVGAQGKAI.GIDKF
Spinach          AGDELRKEGKAVRVVSFVSWELFEKQSDEYKESVLPSDVTARVSIEAGSTFGWHKIVGSKGKAI.GIDKF
Yeast            AAKTLAAKNIKARVVSLPDFFTFDKQPLEYRLSVLPDNV.....PIMSVEVLATTCWGKYAHQSFGIDRF
Mouse            RVLDPFTIKPLDRKLILDSARATKG.RILTVEDH....YYEGGIGEAVS.AAVVGEPGVTVTRL....AV
Rat              RVLDPFTIKPLDKKLILDCARATKG.RILTVEDH....YYEGGIGEAVS.AVVVGEPGVTVTRL....AV
Human            RVLDPFTIKPLDRKLILDSARATKG.RILTVEDH....YYEGGIGEAVS.SAVVGEPGITVTHL....AV

                            680       690       700
Mtuberculosis    GESADHKTLFREYGFTAEAVAA...AA......ERALDN........
MbovisBCG        GESADHKTLFREYGFTAEAVAA...AA......ERALDN........
Mleprae          GESADYQTLFREYGFTPEAVVA...AA......EQVLDN........
Mmarinum         GESADYKTLFREYGFTAEAVAA...AA......ERALEN........
Msmegmatis       GESADDKTLFREFGFTPEAVAA...AA......ERSLEN........
Cglutamicum      GASADYQTLFEKFGITTDAVVA...AA......KDSING........
Ecoli            GESAPAELLFEEFGFT..VDNV...VA......KAKELL........
Banthracis       GASAPGEKIMEEYGFTVENVVR...KV......KEML..........
Bsubtilus        GASAPGETIINEYGFSVPNVVN...RV......KALINK........
Staphylococcus   GASAPGDLVVEKYGFTKENILNQ..VM......SL............
Maize            GASAPAGTIYKEYGITVESIIA...AA......KSF...........
Spinach          GASAPAGKIYQEYGITVEAVVE...AA......KSVC..........
Yeast            GASGKAPEVFKFFGFTPEGVAE...RAQKTIAFYKGDKLISPLKKAF
Mouse            SQVPRSGKPAELLKMFGIDKDAIVQAV......KGLVTKG.......
Rat              SQVPRSGKPAELLKMFGIDKDAIVQAV......KGLVTKG.......
Human            NRVPRSGKPAELLKMFGIDRDAIAQAV......RGLITKA.......
```
